# HDL from 36-h fasted participants potently promotes efflux of cholesteryl ester from activated microglia

**DOI:** 10.3389/fnagi.2025.1629496

**Published:** 2025-08-26

**Authors:** Joanne K. Agus, Oscar M. Muñoz Herrera, Christopher H. Rhodes, Jack Jingyuan Zheng, Chenghao Zhu, Maurice Wong, Xinyu Tang, Izumi Maezawa, Lee-Way Jin, Carlito B. Lebrilla, Danielle J. Harvey, Angela M. Zivkovic

**Affiliations:** ^1^Department of Nutrition, University of California, Davis, Davis, CA, United States; ^2^Department of Pharmacology and Toxicology, University of California, Davis, Davis, CA, United States; ^3^Department of Biochemistry and Molecular Medicine, University of California, Davis, Davis, CA, United States; ^4^Department of Pathology and Laboratory Medicine, University of California Davis Medical Center, Davis, CA, United States; ^5^Department of Public Health Sciences, University of California, Davis, Davis, CA, United States

**Keywords:** high-density lipoprotein, dementia, Alzheimer’s disease, fasting, cholesterol accumulation

## Abstract

The potential impact of lifestyle changes such as prolonged fasting on brain health still remains unclear. Neurodegenerative diseases often exhibit two key hallmarks: accumulation of misfolded proteins such as amyloid beta oligomers (AβO) and intracellular cholesterol accumulation. In this study, we investigate how a 36-h fast affects the capacity of isolated high-density lipoproteins (HDLs) to modulate the effects of AβO and excess cholesterol in microglia. HDL from 36-h fasted individuals were significantly more effective in effluxing cholesteryl esters from treated microglia, showing a remarkable 10-fold improvement compared to HDL from the postprandial state. Furthermore, the ability of 36-h fasted HDL to mitigate the reduction of apolipoprotein E secretion in AβO- and cholesterol-loaded microglia surpassed that of postprandial HDL. In exploring differences among HDL parameters from postprandial, overnight fasted, and 36-h fasted individuals, we observed that plasma HDL-cholesterol and apolipoprotein A-I concentrations remained unchanged. However, nuclear magnetic resonance (NMR) analysis revealed reduced total HDL particle count, a decrease in the smallest HDL particles (HDL1, 7.4 nm diameter), and an increase in the largest HDL particles (HDL7, 12 nm) after the 36-h fast. Transmission electron microscopy (TEM) analysis further found an increase in even larger HDL particles (12–14 nm) in 36-h fasted individuals. Targeted mass spectrometry (MS)-based proteomics and glycoproteomics unveiled a reduction in HDL-associated apolipoprotein A-IV and disialylated apolipoprotein C-III content following the 36-h fast. These findings collectively suggest that prolonged fasting induces structural, compositional, and functional alterations in HDL particles, and influences their capacity to attenuate the effects of excess cholesterol and AβO in microglia.

## 1 Introduction

Alzheimer’s disease (AD) is the most common cause of dementia, affecting an estimated 6.9 million people aged 65 and older in the United States in 2024, making it a growing public health concern (Alzheimers Dementia, 2024). In addition to the well-known amyloid beta (Aβ)- and tau-related pathological hallmarks of AD (Heneka et al., 2015), intracellular lipid deposition is characteristic of AD. Excessive storage of cholesteryl esters (CE) has been found in the AD brain and microglia (Foley, 2010; Zareba and Peri, 2021), Microglia, specialized phagocytic cells of the brain, play a critical role in the clearance of Aβ, which is involved in the pathogenesis of AD (Ries and Sastre, 2016; Tarasoff-Conway et al., 2015), The ability of microglia to remove Aβ is influenced by their cellular cholesterol clearance capacity (Shibuya et al., 2015).

High-density lipoproteins (HDLs) play a critical role in reverse cholesterol transport (RCT) and have recently been shown to perform an array of other essential functions, including regulation of immune function (Marsche et al., 2013; Soran et al., 2012; Wei et al., 2020; Zhu and Parks, 2012), and inflammation (Marsche et al., 2013; Zheng et al., 2021; Zhu et al., 2019b). HDL particles exert their immunoregulatory and anti-inflammatory effects in part by modulating cholesterol content in plasma membranes (Juhl and Wüstner, 2022; Wei et al., 2020; Zhu and Parks, 2012), as well as lipid droplets and cellular organelles, in a complex process involving multiple pathways (Juhl and Wüstner, 2022). In microglia, intracellular Aβ degradation is mediated by the cholesterol efflux function via apolipoprotein E (ApoE) (Lee et al., 2012), which is induced by HDL particles. In neurodegenerative conditions, microglia accumulate cholesterol and lipid-rich debris (Zareba and Peri, 2021). The extent to which Aβ impairs microglial cholesterol efflux to cholesterol acceptors (i.e., HDL), especially in the context of intracellular cholesterol accumulation, remain unknown.

Interest in HDL particles in the context of AD has been growing given recent findings linking HDL with protection from AD in humans, and with reversal of neurodegeneration in animal models. High plasma HDL-C is associated with a strong reduction in AD risk (HR 0.4) (Zhu and Parks, 2012), conversely, AD patients have low concentrations of apolipoprotein A-I (ApoA-I), the defining HDL protein (Luo et al., 2017). Mice injected with reconstituted HDL peripherally experienced reductions in brain soluble Aβ peptides (Yassine et al., 2015), and overexpression of peripheral ApoA-I reduced brain Aβ burden, reduced neuroinflammation, and preserved cognitive function (Qu et al., 2019). However, simply raising HDL-C is not a safe strategy for improving outcomes related to HDL. HDL-C concentrations have been found to have a U-shaped curve with respect to mortality outcomes, with both low (<30 mg/dl) and high (>100 mg/dl) HDL-C being associated with increased risk of mortality (Madsen et al., 2017). Recent studies have also uncovered an increased risk for dementia in individuals with very high HDL-C (>85 mg/dl), highlighting the need for analyses beyond the simple measurement of HDL-C to understand how HDL particles may protect against neurodegenerative disease (Hussain et al., 2024), such as HDL functionality [i.e., cholesterol efflux capacity (CEC)] and structure (i.e., size distribution and lipoprotein glycosylation).

In the context of AD, patients were found to have reduced HDL functionality, i.e., lower CEC (Marsillach et al., 2020). Evidence shows that both the functionality and structure of HDL particles is affected by multiple factors, including particle composition. The ability of HDL to perform cholesterol efflux and regulate inflammation and immunity is affected by their lipid, protein, and glycan cargo (Soran et al., 2012; Zheng et al., 2021; Zhu et al., 2019b). Our team has demonstrated that HDL-associated proteins are differentially N- and O-glycosylated compared to those same proteins in plasma (Kailemia et al., 2018), and that HDL glycoprofiles can be predictive of immunomodulatory capacity (Krishnan et al., 2017).

In terms of HDL structure, clinical studies show that HDL size distribution is strongly associated with several metabolic conditions, such as cardiovascular disease (German et al., 2006; Kontush, 2015; Watanabe et al., 2006). However, different HDL subpopulations are associated with different diseases, for example, small HDL are associated with chronic kidney disease and large HDL are associated with proteinuria (Alabakovska et al., 2002; Honda et al., 2016; Kontush, 2015; Soto-Miranda et al., 2012). While it is clear that somehow improving HDL is an important therapeutic strategy for decreasing the risk of chronic diseases such as cardiovascular disease and AD, it is not clear exactly what aspect of HDL should be altered to improve outcomes, and how.

We and others have previously shown that the HDL proteome, lipidome, and functional capacity can be remodeled through diet (Bardagjy and Steinberg, 2019; DiMarco et al., 2017; O’Reilly et al., 2016; Zhu et al., 2019a), and that these alterations can be achieved within a relatively short period of time (Hong et al., 2022; Sawrey-Kubicek et al., 2019; Zhu et al., 2019b). Fasting shows therapeutic promise for reducing risk factors in vascular dementia, AD, and cognitive impairment, due to its ability to modulate neuroinflammation, synaptic disfunction, neurovascular function, oxidative stress, and beta-amyloid accumulation (Elias et al., 2023; Tagliafico et al., 2023; Yoon and Song, 2019). However, all studies highlighted the need for further validation and studies in diverse cohorts of human participants, especially with regard to the safety and tolerability of fasting for certain vulnerable populations such as older individuals with frailty. Additionally, comparatively little is known about the effects of prolonged fasting on HDL. Several studies have shown that different forms of decreasing meal frequency, from intermittent fasting to time-restricted eating patterns, can lead to increases in HDL-C, improvements in CEC (Lamine et al., 2006), and alterations in HDL particle size distribution (Bhutani et al., 2013; Grundler et al., 2021; Rhodes et al., 2023). Additionally, a recent study showed that fasting-mimicking diets can reduce neuroinflammation in mice (Rangan et al., 2022).

In this study, we aimed to investigate the effects of prolonged fasting on HDL functionality (i.e., CEC), and structure (i.e., size distribution and lipoprotein glycosylation). Additionally, we examined whether these changes mitigate cholesterol and CE efflux in amyloid beta oligomer (AβO) and cholesterol-loaded microglia.

## 2 Results

All of the participants were determined to be compliant for fasting throughout the 36 h, as monitored by blood glucose readings and ketone body concentrations (Rhodes et al., 2023). Participants also consumed the same foods and beverages on day 1 and day 3 of the study, as per the study design (Rhodes et al., 2023).

### 2.1 Effects of fasted vs. postprandial HDL on microglia cholesterol content and apolipoprotein E secretion

We challenged human microglia clone 3 (HMC3) microglia with cholesterol (Chol), AβO, or cholesterol + AβO (Chol + AβO) to investigate how microglia Chol handling is affected by these treatments, simulating the environment in the AD brain. We also investigated the effects of adding HDL particles from individuals in the fed and fasted states.

**FIGURE 1:**
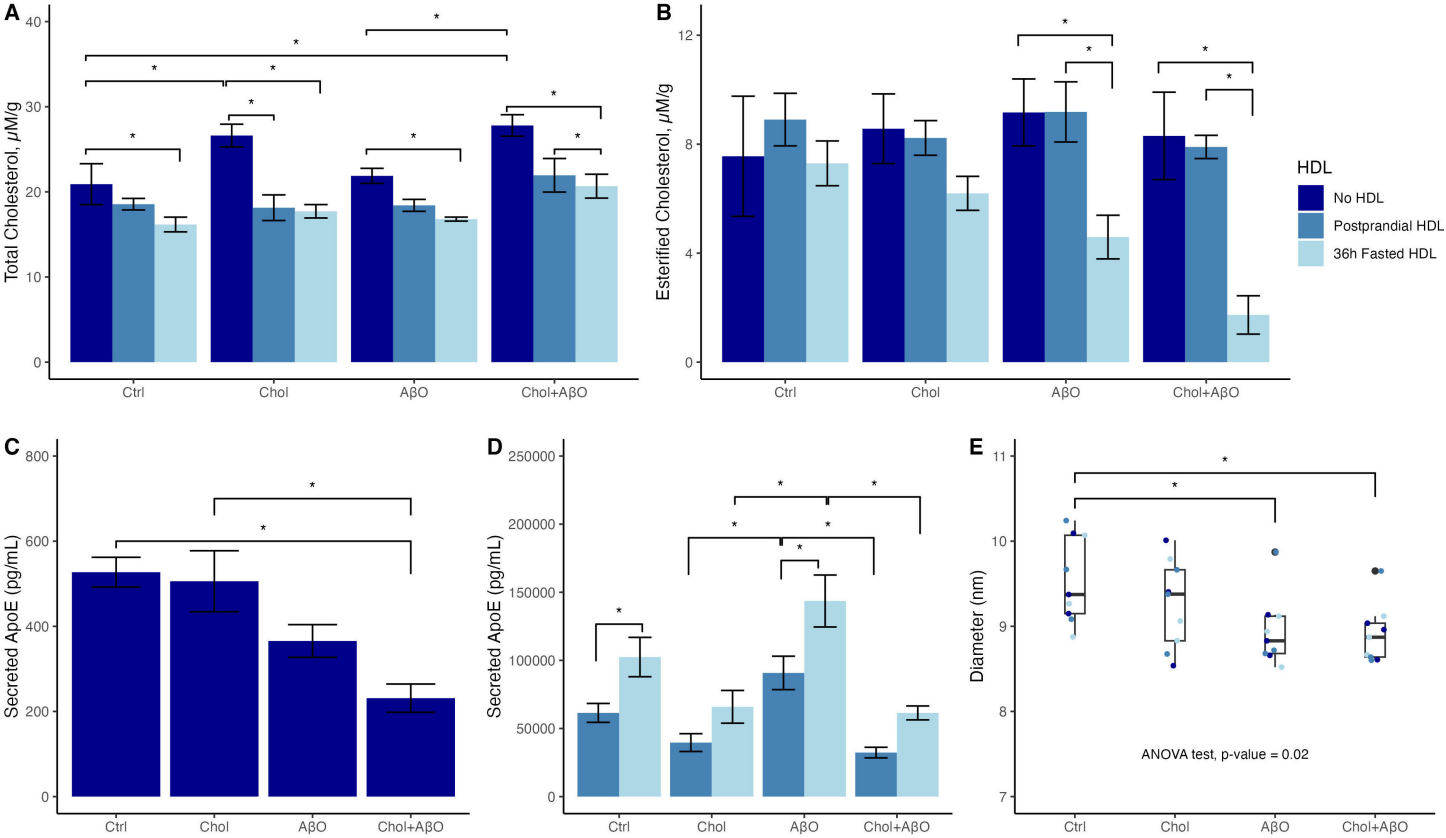
The effects of Chol, amyloid beta oligomer (AβO) or Chol + AβO vs. control, and postprandial or 36 h fasted HDL vs. no HDL on HMC3 microglia: **(A)** total cellular cholesterol, and **(B)** esterified cholesterol content. Effects of Chol, AβO or Chol + AβO on: **(C)** apolipoprotein E (ApoE) secreted into the supernatant with no HDL incubation, **(D)** with fed vs. fasted HDL incubation. **(E)** Diameter of particles secreted into the supernatant. Shown are means ± SE (*n* = 3). *P* values < 0.05 were noted with asterisk (*).

Cholesterol and Chol + AβO treatment but not AβO alone increased total cellular Chol concentrations compared to control, with Tukey adjusted *p* values of 0.0237 for Chol vs. Ctrl and 0.0053 for Chol + AβO vs. Ctrl (Figure 1A and Supplementary Table 1). In all three treatments as well as in the control cells, adding 36 h fasted HDL significantly attenuated the Chol increase, whereas postprandial HDL decreased cellular Chol in Chol (Tukey adjusted *p*-value = 0.0003) and Chol + AβO treated cells (Tukey adjusted *p*-value = 0.011) but not in control and AβO treated cells (Figure 1A). Strikingly, only 36 h fasted but not postprandial HDL was capable of reducing the cellular content of CE in AβO (Tukey adjusted *p*-value = 0.023) and Chol + AβO (Tukey adjusted *p*-value = 0.0012) treated cells, with the strongest effect observed in the Chol + AβO cells (Figure 1B). The amount of ApoE secreted into the supernatant was significantly reduced in Chol + AβO cells (Tukey adjusted *p*-value = 0.0095, Figure 1C and Supplementary Table 2), suggesting that at least part of the observed accumulation of cellular Chol was due to decreased efflux via ApoE. Again, 36 h fasted HDL were significantly better at stimulating ApoE secretion than postprandial HDL, and this effect was strongest in AβO treated cells (*p*-value = 0.0041, Figure 1D and Supplementary Table 2). The diameter of secreted ApoE particles in the supernatant was significantly reduced in Chol + AβO treated cells (Tukey adjusted *p*-value = 0.031, Figure 1E and Supplementary Table 3), suggesting that when microglia are challenged with Chol + AβO their Chol efflux is impaired not only because they secrete less ApoE but also because they secrete smaller particles, which carry less Chol per particle.

### 2.2 Changes in lipoprotein particle size distribution

In exploring differences among HDL parameters from postprandial, overnight fasted, and 36-h fasted individuals, we observed that plasma HDL-cholesterol remained unchanged (Rhodes et al., 2023). To investigate the factors contributing to differences in CEC between HDLs from postprandial and 36-h fasted individuals, we compared the lipoprotein size distribution across all timepoints.

**FIGURE 2:**
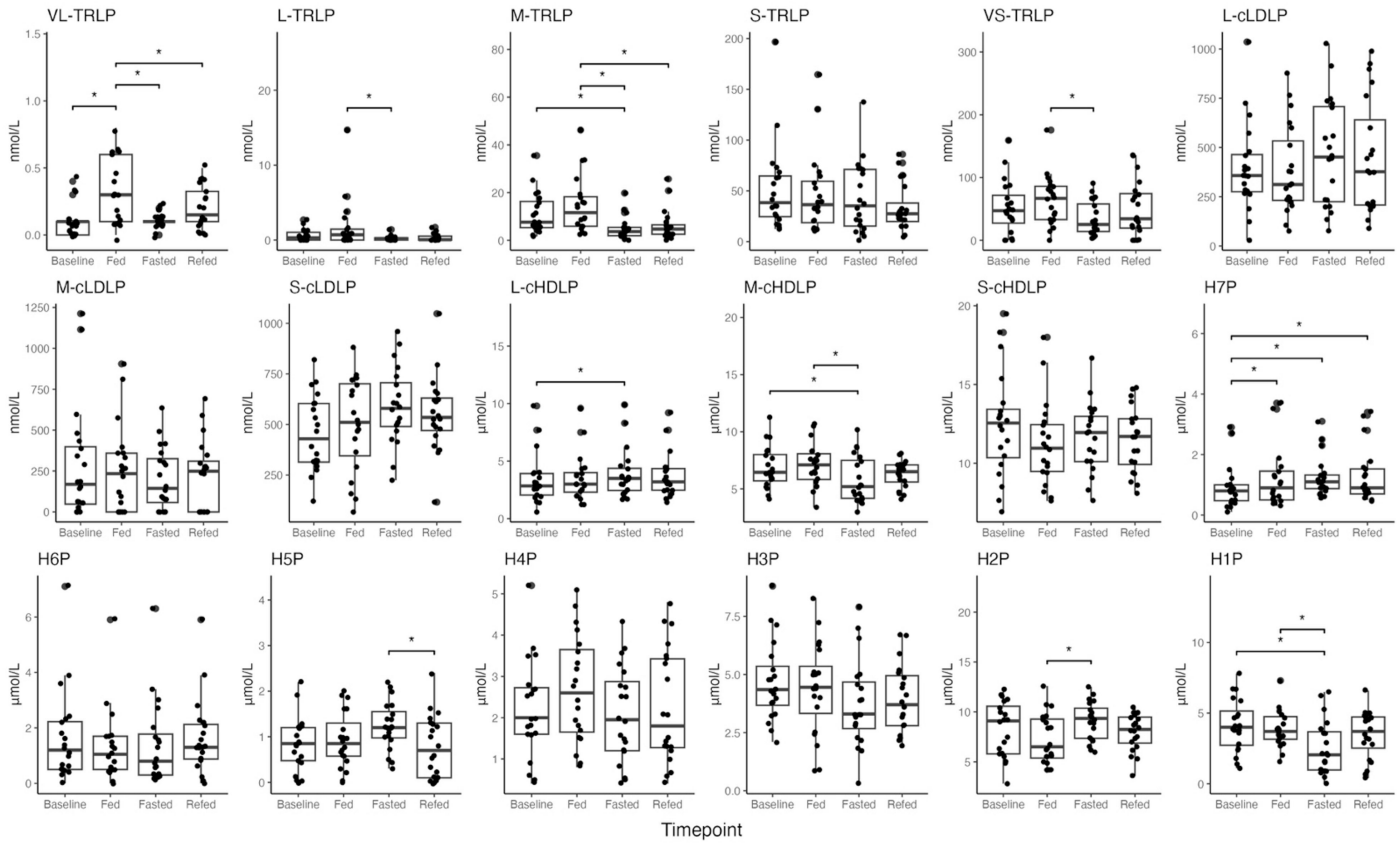
Boxplots of NMR lipoprofile data of high-density lipoprotein particles (HDLP), triglyceride-rich lipoprotein particles (TRLP), and low-density lipoprotein particles (LDLP), including their subtypes (large, medium, small, and H1P–H7P) across all timepoints (Baseline, Fed, Fasted, Refed). Description of size differences between lipoprotein subtypes is provided in Supplementary Table 7. *P* values < 0.05 were noted with asterisk (*). Differences between timepoints were verified using linear mixed effect models.

Nuclear magnetic resonance (NMR) lipoprofile analysis was performed to determine both the changes in overall lipoprotein particle size distribution across all lipoprotein classes, and to determine the specific changes within HDL particles of prolonged fasting. ANOVA analysis in Supplementary Table 4 shows that concentrations across HDL subclasses were significantly different across fasting timepoints, with the greatest difference observed in particle concentrations of subclass H7P (HDL particles size 12 nm in diameter, adjusted *p*-value = 0.017), H1P (HDL particles size 7.4 nm in diameter, adjusted *p*-value = 0.020 adjusted), H2P (HDL particles size 7.8 nm in diameter, adjusted *p*-value = 0.032), M-cHDLP (medium calibrated HDL particles, size 8.1–9.5 nm in diameter, adjusted *p*-value = 0.020), and L-cHDLP (large calibrated HDL particles 9.6–13 nm in diameter, adjusted *p*-value = 0.032). These differences are mostly driven by comparing HDL particle concentrations between the Baseline and Fasted timepoints (Figure 2 and Supplementary Table 4). Between these two timepoints, pairwise comparison revealed a decrease in particle concentration of H1P (adjusted *p*-value = 0.017) and M-cHDLP (adjusted *p*-value = 0.026), and an increase in particle concentrations of L-cHDLP (large calibrated HDL particle size 9.6–13 nm in diameter, adjusted *p*-value = 0.017) and H7P (adjusted *p*-value = 0.017) (Figure 2 and Supplementary Table 4). Changes in concentrations of HDL particles were not significant when comparing the Fed and Refed states (Figure 2 and Supplementary Table 4).

Concentrations of triglyceride rich lipoprotein (TRLP) were also altered across timepoints, and mostly driven by changes between the Fasted and Fed states, and not between the Fasted and Baseline states (Figure 2 and Supplementary Table 4). There were, however, a few significant changes observed in TRLP concentrations between the Fasted and Baseline states, which include a significant increase in S-cLDLP concentration (small calibrated LDL particles, size 19–20.4 nm in diameter, adjusted *p*-value = 0.032, Figure 2 and Supplementary Table 4), and a decrease in M-TRLP concentration (medium TRLP, adjusted *p*-value = 0.017, Figure 2 and Supplementary Table 4). No other changes in TRLP between Baseline and Fasted were observed. Significant changes observed in the concentration of very large TRLP (VL-TRLP, adjusted *p*-value = 0.0005) were driven by an increase in particle concentration at the Fed compared to Baseline and the Fasted states (adjusted *p*-value = 0.0004, adjusted *p*-value = 0.0006, Figure 2 and Supplementary Table 4), but no significant differences were found when comparing VL-TRLP in the Refed to Baseline and the Fasted states (adjusted *p*-value = 0.348, adjusted *p*-value = 0.168, Figure 2 and Supplementary Table 4).

Additionally, no significant differences in lipoprotein concentration were observed between the Fasted and Refed states (Figure 2 and Supplementary Table 4). No significant differences were observed when comparing the Refed and Fed states, except for the decrease in particle concentration of M-TRLP (medium TRLP, particle size 37–49 nm in diameter, adjusted *p*-value = 0.003, Figure 2 and Supplementary Table 4).

**FIGURE 3:**
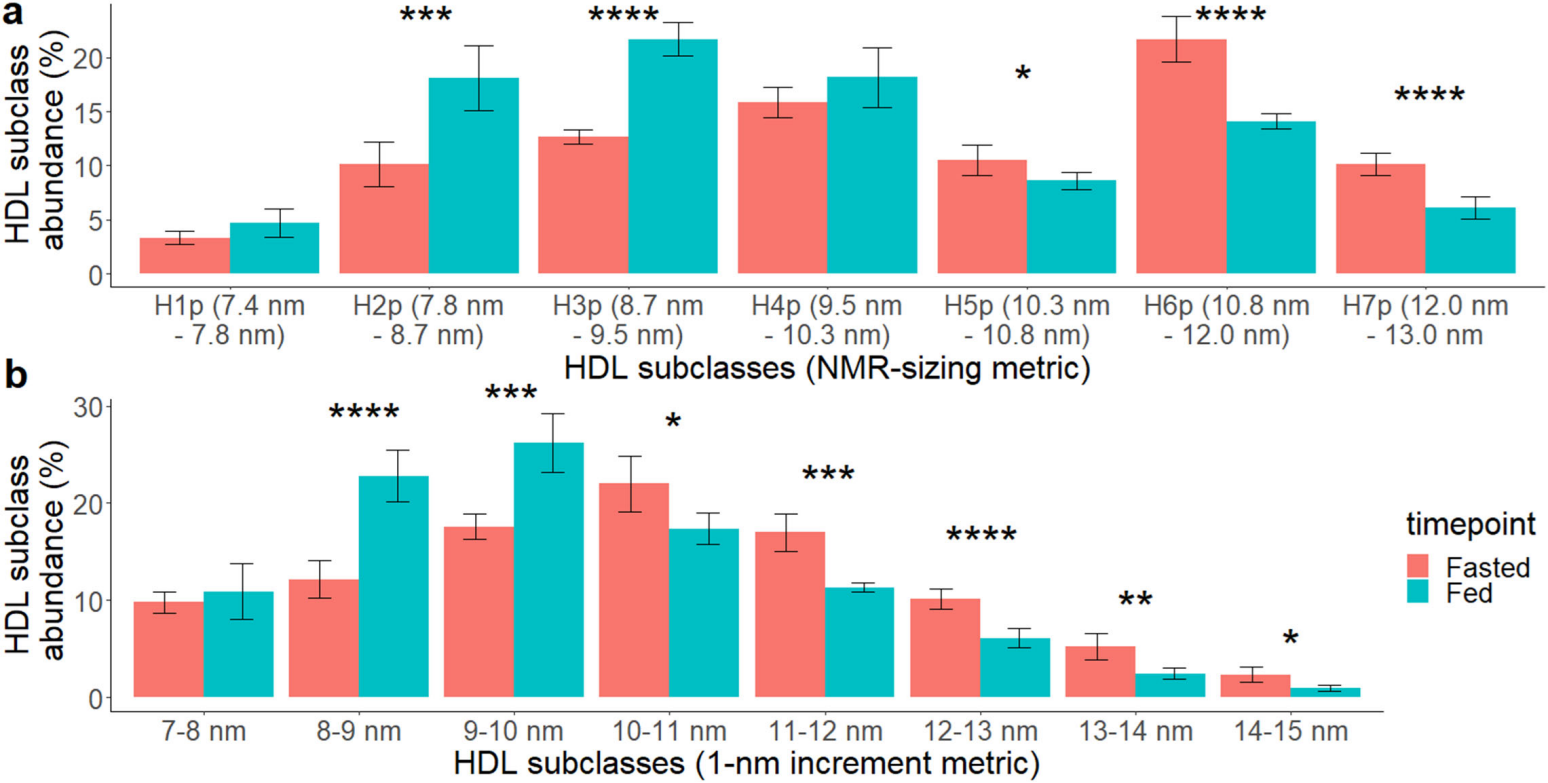
Effects of 36 h of fasting on HDL particle size distribution comparing the fasted (red) vs. the fed (turquoise) state. HDL particle size (diameter) was measured using transmission electron microscopy (TEM) and the continuous particle size data are displayed using **(a)** NMR lipoprofile HDL subclass size ranges and **(b)** 1-nm increment size ranges. The following *P* values were noted with asterisk: < 0.05 (*). < 0.01 (**), < 0.001 (***), and < 0.0001 (****).

The changes in HDL size distribution between fed and 36 h-fasted states were further verified by transmission electronic microscope (TEM) and compared the results from TEM to those from NMR. According to TEM, the average particle size of the pooled fasted and postprandial HDL showed that the mean diameter of the fasted HDL particles (10.73 nm ± 2.53 nm) was significantly increased compared to the post-prandial HDL (9.93 nm ± 1.93 nm, *p* < 0.001) (Supplementary Figure 1). We further classified HDL particles into subclasses by their diameters using a 1-nm-increment metric. These measurements were compared with those obtained using the NMR sizing metric. The detailed distribution changes of HDL particle size in the fed and fasted states are shown in Figure 3. In the NMR-sizing metric, there were more small HDL particles in the fed-state [H2P (7.8–8.7 nm, 18.1%), H3P (8.7–9.5 nm, 21.7%)] compared to that of the fasted state (H2P: 10.1%, *p* = 0.002, H3P: 12.7%, *p* < 0.001, Figure 3a), while there were more large HDL particles in the fasted state [H5P (10.3–10.8 nm, 10.5%), H6P (10.8–12.0 nm, 21.7%), and H7P (12.0–13.0 nm, 10.2%)] compared to that in the fed state (H5P: 8.6%, *p* = 0.03, H6P: 14.1%, *p* < 0.001, H7P: 6%, *p* < 0.01, Figure 3a). The 1-nm-increment metric showed the same HDL size distribution pattern between the fed and the fasted states (Figure 3b). TEM images of isolated HDL particles can be found in Supplementary Figure 2.

### 2.3 HDL glycoproteomic and proteomic changes

**FIGURE 4:**
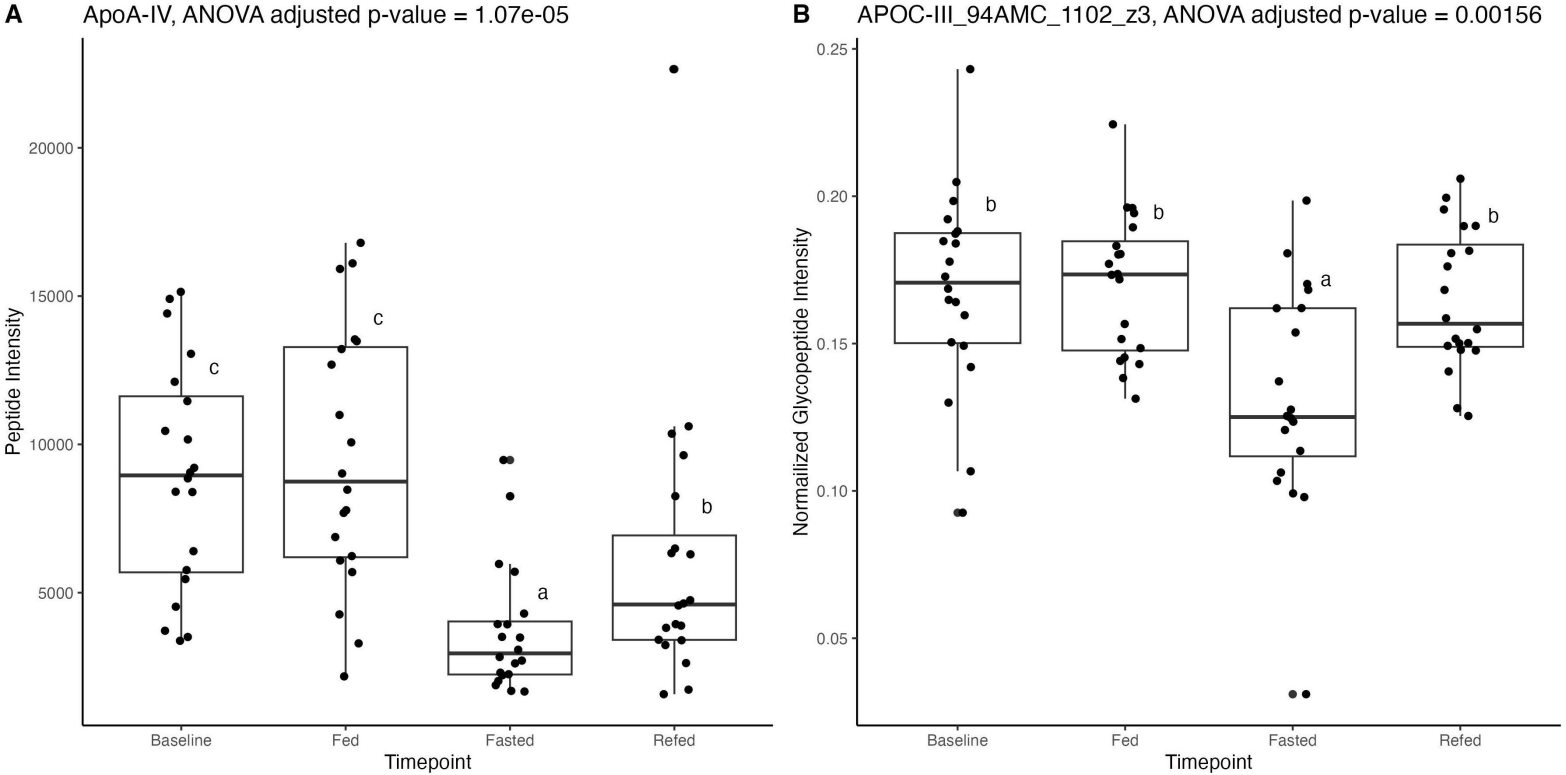
**(A)** Proteomic data analyzed with dynamic multiple reaction monitoring mass spectrometry-based method showing significant reduction in apolipoprotein A-IV (ApoA-IV) peptide in the Fasted state, after 36 h of fasting, compared to the Baseline state, after 12 h of fasting. **(B)** Glycoproteomic data analyzed with ultra performance liquid chromatography-triple quadrupole mass spectrometry showing significant reduction in apolipoprotein C-III (ApoC-III) peptide at position 94 in the Fasted state, after 36 h of fasting, compared to Baseline, after a standard overnight 12 h fast. Differences between timepoints were verified using linear mixed effect models. Superscript letters (a–c) indicate statistical comparisons; bars sharing the same letter are not significantly different (*P* ≥ 0.05), while bars with different letters differ significantly (*P* < 0.05).

Proteomic and glycoproteomic analyses were performed to identify changes in HDL-associated proteins and glycoproteins in the overnight fasted, 36-h fasted, and postprandial states. Proteomic data revealed that while most HDL-associated proteins were unchanged, apolipoprotein A-IV (ApoA-IV) concentrations were significantly reduced in the fasted compared to baseline states (*p*-value = 3.34e-7, adjusted *p*-value = 2.16e-5) and also in the Fed compared to the Refed state (*p*-value = 0.004, adjusted *p*-value = 0.14) (Figure 4A and Supplementary Table 5). Out of 32 HDL-associated proteins, 45 peptides and 97 glycopeptides were monitored. After correction for multiple testing, the relative concentration of the disialylated glycopeptide 1,102 at site 94 of apolipoprotein C-III (ApoC-III) was significantly reduced in the 36-h fasted compared to the overnight fasted (*p*-value = 8.56e-5, adjusted *p*-value = 0.0078), Fed (*p*-value = 7.19e-5, adjusted *p*-value = 0.0033), and Refed states (*p*-value = 0.000124, adjusted *p*-value = 0.0038) (Figure 4B and Supplementary Table 6), however the concentration of ApoC-III remained unchanged (*p*-value = 0.74, Supplementary Table 5).

## 3 Discussion

Our study investigated the potential protective effects of HDL particles against AD progression by examining their capacity to efflux cholesterol from microglia exposed to cholesterol and AβO. By comparing the CEC of HDLs isolated from postprandial and 36-h fasted individuals, we found that HDLs from both conditions attenuated the cholesterol increase, and 36 h of fasting is a physiologically potent intervention to alter HDL particles (HDLP) function and profile when compared to 12 h baseline fasted and post-prandial time points. We further explored factors contributing to the differences in efflux capacity among HDL particles derived from different conditions. Our results revealed an overall increase in average HDLP size after 36 h of fasting, as demonstrated by both NMR and TEM analyses. Additionally, HDLs from the 36-h fasting condition showed reduced levels of ApoA-IV and disialylated ApoC-III compared to other conditions.

Even though cellular levels of CE were not significantly increased in HMC3 microglia by treatment with Chol, AβO, or Chol + AβO, the 36 h fasted HDL were able to efflux CE, which is only found in lipid droplets intracellularly, from AβO and Chol + AβO treated microglia to concentrations well below all control conditions. This is of importance because one group has reported that peripheral ApoA-I HDL can penetrate the blood–brain barrier (BBB) through the scavenger receptor class B type I (SR-BI)-mediated transcytosis system (Bhutani et al., 2013; Fung et al., 2017), which suggests the possibility that peripheral HDL may interact with brain cells such as microglia to efflux excess intracellular cholesterol, affecting their function and phenotype. Cholesterol handling in microglia is essential because high cholesterol concentrations impair the ability of microglia to clear Aβ, and increase microglial inflammatory signaling and ROS production (Grundler et al., 2021; Muñoz Herrera and Zivkovic, 2022). All of this further drives the accumulation of Aβ oligomers and eventually plaque formation, as well as creating a pro-inflammatory environment that contributes to neurodegeneration (Baerends et al., 2022; Fung et al., 2017; Grundler et al., 2021; Guglielmotto et al., 2014; Muñoz Herrera and Zivkovic, 2022; Muñoz Herrera and Zivkovic, 2022; Rangan et al., 2022; Tamagno et al., 2006). HDL can be protective against the progression of AD in the brain by modulating Aβ synthesis by regulating cholesterol concentrations in the cell; this, in turn, enhances Aβ clearance and degradation (Jin et al., 2021; Wang et al., 2021). Future studies are needed to determine the effects of the full range of fasting durations (from overnight fasts, to 16 h fasts typical of intermittent fasting regimens, to 24 h fasts, to multiple day fasts) on the CEC of HDL, particularly in the context of its effects on brain cells. These questions hold translational relevance, as they may inform the development of lifestyle-based interventions aimed at modulating HDL function to promote neuroprotective cholesterol handling in the brain.

To further elucidate HDL’s modulation ability, we reported that changes to HDL function at 36 h fasted is also associated with significant changes in HDLP size distribution. These differences were driven by the increase in particle concentration of L-HDLP and decrease in particle concentration of M-HDLP and S-HDLP. However, studies on the effects of fasting on HDL size distribution are limited (Bhutani et al., 2013; Grundler et al., 2021). Only two studies report changes in HDL size distribution following fasting intervention, one including alternate day fasting and exercise intervention (Bhutani et al., 2013) and one as part of a modified prolonged fasting and lifestyle intervention (Grundler et al., 2021). Both studies reported a decrease in concentration of small HDLP and an overall increase in average HDLP size. Our findings also report a decrease in concentration of small HDLP, including HDLP subtype H1P and H2P (particle size 7.4 and 7.8 nm in diameter), but we additionally report an increase in HDLP subtype H7P and L-cHDLP (particle size 12 nm, and 9.6–13 nm) as measured by NMR lipoprofile analysis. This shift in distribution increases the average HDLP size and suggests a shift in distribution of HDL subtype diversity and function (Jomard and Osto, 2020).

Particles analyzed from TEM micrographs provide size information for every particle, allowing us to estimate the abundance of particles within specific size ranges. We applied the size cutoffs used in NMR analysis on TEM-generated HDL particle size data. We compared data from Figures 2, 3: for the size groups of H1P, H2P, H3P, H4P, H5P, and H7P the direction of change of particle abundance agrees between the two analyses. For H6P, the changes of particle abundance were opposite between the two analyses, though the differences observed in both are not statistically significant. Generally, both HDLP sizing methods agree that HDLP from the fasted state have decreased small HDLP and increased large HDLP abundances. Whereas the NMR analyses measured the changes to HDL particle size distribution in unprocessed plasma, TEM was used to confirm changes in processed, isolated HDL from plasma in order to fully understand the particle size distribution of the HDL particles that were used in the *in vitro* assays and to confirm that these accurately reflected the observed changes to HDL particle size distribution in unprocessed plasma.

Both NMR lipoprofile and TEM results highlight that HDLP size changes are more sensitive than measuring HDL-C alone in the acute prolonged fasting state. Other studies have shown that HDLP number, size, and function are associated with cardiovascular disease, coronary artery disease, and all-cause mortality more strongly than HDL-C concentration (Cheng et al., 2022; Kontush, 2015; Rohatgi et al., 2014). This suggests that HDLP size distribution could represent a unique function and composition of particles that are specific to a certain health or disease state (Fazio and Pamir, 2016). Unfortunately, the relationship between HDLP size and function is still unclear. Some studies have found that small HDLP are associated with higher CEC than larger HDLP (Du et al., 2015), but not in all cases (Mutharasan et al., 2017). This highlights the importance of evaluating the context at which these HDLP profiles change to further understand how size, function, and risk are associated with each other. For instance, our study reported that compared to Baseline and Fed states, 36 h of fasting increased average HDLP size by increasing the abundance of large HDLP and decreasing the abundance of small and medium HDLP, and induced a higher efflux of CE from activated microglia, suggesting that 36 h fasted large HDLP may be functionally and compositionally different from other large HDLP from other physiological states. Future studies in a variety of disease conditions and healthy states should examine this relationship further to answer remaining key questions in HDL biology including whether small, medium and large HDL particles are defined only by size or also by additional differences in composition and function. For example, it is entirely possible that small HDL in one physiological state are more functional and less deleterious than small HDL in another condition. With quadrillions of HDL particles circulating per milliliter of plasma, it is easy to imagine that even more refined subclass categorization beyond what has been achieved thus far (based on size alone, apoprotein content alone, charge and size, or density ± size) will be required for a complete understanding of HDL biology.

We and others have previously reported that proteomic analysis of isolated HDL fractions shows an increased abundance of transfer proteins such as LCAT and PLTP in large HDL particles, enhancing cholesterol esterification and core lipid storage (Davidson et al., 2022; Zheng et al., 2021). While the NMR data indicate a significant increase in the concentration of large HDL particles, the proteomic data revealed no changes in HDL-associated proteins between fasting and postprandial states, except for a decrease in ApoA-IV. ApoA-IV is primarily expressed in the upper intestine in humans, (Kohan, 2015; Qu et al., 2019) with very little expression detected in the liver (Karathanasis et al., 1986). Although most circulating ApoA-IV has been detected in the plasma fraction with a density > 1.21 g/ml (Ghiselli et al., 1986; Kohan, 2015; Zheng et al., 2021), it is enriched in small HDL (HDL3) (Andraski et al., 2023) particles and also colocalizes with apolipoprotein B (ApoB) containing and TRLP (i.e., chylomicrons) from the intestine (Kohan, 2015). Han et al. (2021) reported that enterically derived HDL harvested from the portal vein are enriched in small HDL (HDL3) particles in mice, although this study did not report on the abundance of ApoA-IV associated with enterically derived HDL, perhaps because in mice, ApoA-IV is expressed abundantly in both the intestinal tract and the liver (Han et al., 2021). Moreover, isotope labeled enteric-HDL have been shown to be detected in plasma as early as 30 min in small HDL particles, and also in large particles at 1.5 h after, indicating rapid (Andraski et al., 2023). It is unclear whether the reduction in the concentration of ApoA-IV in this study reflects fewer ApoA-IV molecules per particle or simply a lower number of ApoA-IV-containing particles. ApoA-IV has been found to participate in the RCT pathway (Peng and Li, 2018), however, whether the depletion of ApoA-IV from HDL particles or the depletion of ApoA-IV-containing particles directly hinders cholesterol efflux function of plasma has not been determined.

Unlike ApoA-IV, ApoC-III concentrations were not significantly altered but the sialylation profiles of ApoC-III were altered across timepoints in isolated HDL particles. ApoC-III associates with all lipoproteins across all sizes and densities, with metabolism and clearance better understood in TRLP (Kailemia et al., 2018; Kegulian et al., 2019; Zheng et al., 2021). ApoC-III sialylation determines its preferential clearance (along with that of the lipoprotein with which it is associated): disialylated ApoC-III_2_ is preferentially cleared via heparan sulfate proteoglycans (HSPG), and monosialylated ApoC-III_1_ is preferentially cleared via LDLR and LRP1 (Kegulian et al., 2019). Reduced ApoC-III_2_ suggests a diminished HSPG-mediated clearance in the fasted state. Because HSPG clearance is slower (Bornfeldt, 2024) than the LDLR and LRP1 clearance pathways (Kegulian et al., 2019), lower ApoC-III_2_ might suggest accelerated removal of the lipoprotein carrying it. However, the relative binding of different ApoC-III glycoforms to HSPG vs. LDLR/LRP1 were observed for TRLP particles, therefore it is unknown if similar differences would be observed for HDL particles. Future studies are needed to explore the effects of differential ApoC-III glycosylation on HDL particle clearance via different pathways. Previously, we demonstrated diet-sensitive shifts in HDL-associated ApoC-III sialylation and O-glycosylation in individuals consuming a fast food vs. a Mediterranean diet (Zhu et al., 2019b). This sensitivity may reflect ApoC-III’s high exchangeability between VLDL and HDL (Ginsberg and Ramakrishnan, 2008). Differences in HDL-associated ApoC-III sialylation status have also been found to be associated with differences in HDL immunomodulatory capacity in stimulated monocytes (Watanabe et al., 2006), and in individuals with vs. without metabolic syndrome (Savinova et al., 2014). Although elevated HDL-ApoC-III concentrations have been shown to reduce HDL CEC (Luo et al., 2017), no studies have yet addressed whether changes in sialylation of ApoC-III in HDL particles affect CEC. Further studies are needed to determine whether changes in HDL ApoC-III glycosylation alter HDL’s immunomodulatory functions, independent of CEC function, particularly in the context of particle size and concentration.

In summary, this study emphasized the potent capacity of HDLs in reducing cholesterol and CE accumulation in activated microglia. The differences in CEC may be driven by HDL size distribution and apolipoprotein composition. Although 36-h fasting significantly enhanced HDL CEC in this study, further validation is needed (Agus and Zivkovic, 2025). The effects of fasting on HDL functionality remain poorly characterized, as most studies report only the effects of fasting on HDL-C and focus on weight loss (Agus and Zivkovic, 2025). Additionally, it is important to note that the findings from this study do not support general recommendations for long-term fasting in all individuals. In the context of early prevention in otherwise healthy individuals, different types of fasting regimens may be a feasible lifestyle intervention to control weight and improve HDL function. However, in older adults, there is a higher risk of malnutrition, muscle wasting, and frailty. Long-term modified fasting regimens such as the Buchinger Fasting Method and the Fasting Mimicking Diet have reported on safety protocols to reduce the potential negative effects of fasting on lean body weight loss (Agus and Zivkovic, 2025). Contraindications to prolonged fasting and recommendations on how to conduct fasting safely have been reviewed, and should be taken into consideration when conducting future studies (Koppold et al., 2024). Additionally, larger studies examining genetic and metabolic heterogeneity in fasting responses are needed to establish its feasibility as a dementia-prevention lifestyle intervention. Future studies should focus on identifying new treatments that can modify HDL functions by altering HDL size distribution, apolipoprotein composition or glycosylation in the context of neurodegeneration.

## 4 Materials and methods

### 4.1 Study design

Plasma samples were obtained from a previously conducted study of a single bout of prolonged fasting in healthy human volunteers (Rhodes et al., 2023). Full clinical study protocols and inclusion and exclusion criteria can be found at clinicaltrials.gov, NCT03487679. Briefly, the study involved 20 participants, 10 males and 10 females, aged 20–40 with no reported health conditions or extreme dietary and exercise patterns. Study participants first came in on day 1 after an overnight fast (12 h) at around 8 a.m. (Baseline: 12 h fasted, timepoint A) for a baseline blood draw, and 2 h after their last meal at around 8 p.m. for their baseline postprandial blood draw (Fed: 2 h postprandial prior to 36 h of fasting, timepoint B). Participants tracked every food and beverage consumed over the entire course of day 1. After the evening meal on day 1 they began their 36-h water-only fast until the morning of day 3, when they came in for a blood draw (Fasted: 36 h fasted, timepoint C) at around 8 a.m. After this blood draw they were instructed to consume the exact same foods and beverages as those they consumed on day 1, and then came in for a final blood draw 2 h after their last meal on day 3 (Refed: 2 h postprandial after 36 h of fasting, timepoint D), again at around 8 p.m. Participants were monitored for fasting compliance with glucose monitoring every 2 h during the study, and ketone body concentrations were later also confirmed. Samples for this secondary analysis were selected based on the significant effect of participant plasma on macrophages observed by Rhodes et al. (2023) between timepoints B and C.

### 4.2 HDL isolation

High-density lipoprotein particles were isolated from plasma using a validated, optimized method (Zheng et al., 2021). Plasma was obtained from a clinical study approved by the UC Davis Institutional Review Board (IRB) (IRB identification [ID], 918915-4). All participants provided informed consent before enrolling in study protocols. The study was conducted in accordance with the Declaration of Helsinki, local legislation and institutional requirements. HDL samples for the *in vitro* experiments were generated from pooled plasma. Plasma from 18 out of 20 of the participants from the original study were included in the plasma pools, as inadequate volumes of plasma remained from 2 of the participants. Equal volumes of total plasma from each individual were combined as described previously (Zheng et al., 2021) to generate a uniform plasma pool for timepoints B and C. This pooled plasma was then aliquoted into 500 μl aliquots, and each aliquot was processed as described previously (Zheng et al., 2021). Briefly, 500 μl starting plasma was thawed and placed under 4.1 ml density solution 1.006 g/ml of potassium bromide (KBr) in 4.7 ml OptiSeal tube (Beckman-Coulter, IN, United States). Then, samples were ultracentrifuged for 30 min at 110,000 RPM using a fixed angle rotor (TLA-110, k factor = 13, Beckman-Coulter). The top 4 ml were removed from the OptiSeal and the remaining 700 μl of sample was mixed with 1.1 ml 1.34 g/ml KBr density solution to create 1.8 ml of a 1.21 g/ml KBr-Lipoprotein and plasma protein fraction. This fraction was pipetted under 2.8 ml of 1.21 g/ml KBr density solution in the OptiSeal tube, and topped off with 100 μl of 1.21 density solution for a second ultracentrifuge spin of 3 h and 30 min at 110,000 RPM. After the second spin, the first 2 ml of the tube was isolated and filtered through 50 KDa Amicon Ultra-4 for 8 min at 4,500 RPM to 250 μl, of which 200 μl was injected into a fast protein liquid chromatograph (FPLC, AKTA P-920) with a size exclusion chromatograph (SEC) column (Superdex 200 GI 10/300), and the system was run at a flow rate of 0.5 ml/min. The HDL fraction was selected based on elution time collecting between the troughs of the LDL-HDL peak and the HDL-albumin peak. The 4 ml of total eluted volume representing the HDL fraction was concentrated using Amicon 50 KDa filters, 2% sucrose as a cryoprotectant (Andraski et al., 2023; Holzer et al., 2017) was added and samples were aliquoted into 20 μl fractions before storage at −80°C. Further pooling was conducted from isolated HDL from each plasma pool isolation of each timepoint to create identical aliquots for *in vitro* experiments.

### 4.3 Proteomic and glycoproteomic analysis

Isolated HDL fractions were analyzed using a previously published method (Tang et al., 2022) which utilizes dynamic multiple reaction monitoring (DMRM) coupled to a Fusion Lumos MS/MS Orbitrap (Thermo Fisher Scientific) for quantifying HDL-associated proteins. Targeted glycoproteomic analysis was conducted using Agilent 1290 Infinity Liquid Chromatography coupled to an Agilent 6495B Triple Quadrupole Mass Spectrometry. A total of 47 peptides and 170 glycopeptides from 33 HDL-associated proteins were monitored, and for each protein a non-glycosylated synthetic peptide was spiked in as a reference standard for quantification. Absolute protein concentrations for apolipoprotein-AI, apolipoprotein-CI, apolipoprotein-D, apolipoprotein-E, and clusterin were determined using a calibration curve. Relative abundances of other peptides were determined by measuring indicator peptides from each protein (Hong et al., 2015; Peng and Li, 2018) and relative abundances of glycopeptides were quantified by the ion count ratio of glycopeptide to indicator peptide of each protein (Hong et al., 2015). Total protein was quantified using the micro BCA kit (Thermo Fisher Scientific, C23235).

### 4.4 NMR lipoprofile analysis

Lipoprotein particle sizes and concentrations were analyzed using proton NMR spectroscopy from whole plasma. NMR lipoprofile is an FDA approved and Clinical Laboratory Standards Institute (CLSI) certified according to guideline EP5-A2 for estimating the concentration of lipoprotein subclasses, which includes LDL particles (LDL-P), HDL particles (HDLP), and VLDL particles (VLDL-P) from small to large, as well as insulin resistance markers (Garcia et al., 2020a; Garcia et al., 2020b). Additional parameters, including ketone bodies and amino acid concentrations were also measured and previously reported (Rhodes et al., 2023).

### 4.5 Microglia experiments

Human microglia clone 3 were cultured using EMEM (ATCC 30-2003), penicillin-streptomycin (10,000 U/ml, Thermo Fisher Scientific, 15140122) and 10% fetal bovine serum (FBS, ATCC 30-2020). HMC3 were seeded on 96-well microplates (Corning; Costar; 3916) 40,000 cells per 200 μl, per well. Seeded cells were allowed to adhere for at least 7 h. Following adherence, media was replaced with EMEM without phenol red, to reduce background signal (Thermo Fisher Scientific, C837K00), penicillin-streptomycin (10,000 U/ml, Thermo Fisher Scientific, 15140122) and 10% FBS (ATCC 30-2020), along with the corresponding treatment.

Dose treatment for both cholesterol and AβO was determined in a previously published paper (Muñoz Herrera and Zivkovic, 2022). Briefly, microglia were loaded with water soluble cholesterol at various concentrations (5, 10, 20, 30, 40, and 50 μg/ml) then assessed using a Green Cytotoxicity Assay (Cat. # G8741) (Results were shown in Supplementary Figure 3a). Further incubation testing was done on AβO treatment at various concentrations (0.1, 0.25, and 0.5 μM) for 24 h. Cytotoxicity of AβO following cholesterol loading was conducted after as well (Results were shown in Supplementary Figures 3b, c) (Muñoz Herrera and Zivkovic, 2022).

Microglia were treated with AβO at 2 μM for 24 h. At the 18 h time-point, the HMC3 were loaded with water soluble cholesterol at 20 μg/ml for 6 h. At the 21 h time-point, the cells were supplemented with HDL at 0.1 mg/ml for 3 h. At 24 h, the supernatant in all wells was removed and stored at −80°C. HDL from 18 out of 20 of the individuals who participated in the study were included due to lack of sufficient material from 2 of the participants. HDL pools from the baseline 2 h postprandial state and the 36 h fasted state were prepared to generate adequate HDL for the *in vitro* experiments. HDL samples from each time point were thawed on ice and 10 μl of each isolate from each participant were taken and combined to generate a pooled HDL sample from each time point with protein concentration determined by micro BCA assay (Thermo Fisher Scientific, C23235). HMC3 cells were lysed using chloroform (Sigma Aldrich, C2432), isopropyl alcohol (Fisher Scientific, 67-63-0) and Np40/Igepal Ca 630 (Sigma Aldrich, 9002-93-1), at 7:11:0.1, respectively. The cellular content of unesterified and esterified cholesterol (CE) was measured using the Total Cholesterol Assay (Cell Biolabs, Inc., STA-390) kit following the manufacturer’s instructions. A portion of the culture supernatant was probed for ApoE content using the Human ApoE ELISA (Cell Biolabs, Inc., STA-367) following the manufacturer’s instructions. The remaining portion of the supernatant was used for analysis of the lipoprotein content, size, and structure via electron microscopy.

### 4.6 Electron microscopy and particle analysis

Transmission electron microscopy analysis was performed as reported (Hong et al., 2022; Zheng et al., 2021) with minor modifications. The detailed protocol and validation of this method have been previously published (Zheng et al., 2025). Briefly, 4 μl samples at 0.1 mg/ml were loaded on a glow-discharged 200-mesh carbon-coated TEM grid (TedPella Inc., CA, United States) for 1 min. The grid was then blot-dried with filter paper and loaded with 2% w/v uranyl formate negative stain solution (pH 4.6), which was quickly blot-dried with a filter paper. The loading and drying of the negative stain solution was repeated 4 more times. After the last negative stain solution was removed and the grid was completely dried, it was stored in a dark environment until sample imaging. Sample grids were imaged using TEM (Talos L120C, Thermo Fisher Scientific) combined with a bottom-mounted CCD camera (Ceta, Thermo Fisher Scientific) at 36,000× magnification and 80 kV electron beam voltage.

Micrographs were processed and particle diameters were obtained using FIJI (Kontush, 2015). Images were processed with the bandpass filter function to filter structures down to 40 pixels and up to 10 pixels. The threshold of the images was then set by the “Moments” mode. The Analyze Particles function was used to obtain particle size in area (nm^2^) and shape information (e.g., circularity, aspect ratio, and roundness), using the following parameters: Size: 19.625–706.5 nm^2^; Circularity = 0.15–1.00; Exclude on edges; Include holes. Selected particle data were then filtered using additional geometrical parameters (aspect ratio < 1.5, roundness > 0.5) to remove non-spherical particles. For data analysis, particle size for each selected particle was reported as nm in diameter, which was calculated by the equation Area/in RStudio (RStudio Team, 2020).

### 4.7 Statistical analysis

Differential abundances of glycopeptides, peptides, and NMR lipoprofile features were performed as linear models (features ∼ Timepoint + Subject (+ batch)) using the limma package in R (R version 4.1.0) (Ritchie et al., 2015). Subjects in the study were used as a fixed effect in the linear models. A one-way ANOVA-like test was used to detect differences between the means of time points. Then the differences between pairs of time points were determined by *post hoc* pairwise comparison. Correction for multiple hypothesis testing was performed using the Benjamini–Hochberg method. The error distribution was tested with the Shapiro–Wilk test. For data violating normality, a log transformation was applied to approach the normal distribution.

Cholesterol concentration, ApoE secretion, and particle sizes were analyzed using linear mixed effect models with the lmerTest and emmeans packages. Two-way ANOVA tests were applied to determine the changes in cholesterol concentration and ApoE secretion according to the treatments and HDL conditions. And a one-way ANOVA test was applied to detect differences in particle sizes in treatments across different HDL conditions. *P*-values of host hoc pairwise comparisons were adjusted using the Tukey’s method.

## Data Availability

All datasets generated and analyzed for this study are included in the article and its Supplementary material. Further inquiries can be directed to the corresponding author.
