# Supplementary material for: HDL from 36-h fasted participants potently promotes efflux of cholesteryl ester from activated microglia
Source: Front Aging Neurosci. 2025 Aug 26;17:1629496. doi: 10.3389/fnagi.2025.1629496 (PMC12417472; doi:10.3389/fnagi.2025.1629496)
Supplement: Supplementary file 1 [file Data_Sheet_1.zip › Microglia Supplemental Material/Supplemetal Content JA 07162025.docx]

HDL from 36h Fasted Participants Potently Promote Efflux of Cholesteryl Ester from Activated Microglia

Joanne K. Agus ^1^ and Oscar M. Muñoz Herrera ^1,2^, Christopher Rhodes ^1^, Jack Jingyuan Zheng ^1^, Chenghao Zhu ^1^, Maurice Wong ^4^, Xinyu Tang ^1^, Izumi Maezawa ^3^, Lee-Way Jin ^3^, Carlito B. Lebrilla ^4^, Danielle J. Harvey ^5^, Angela M. Zivkovic ^1^

**Supplemental materials**


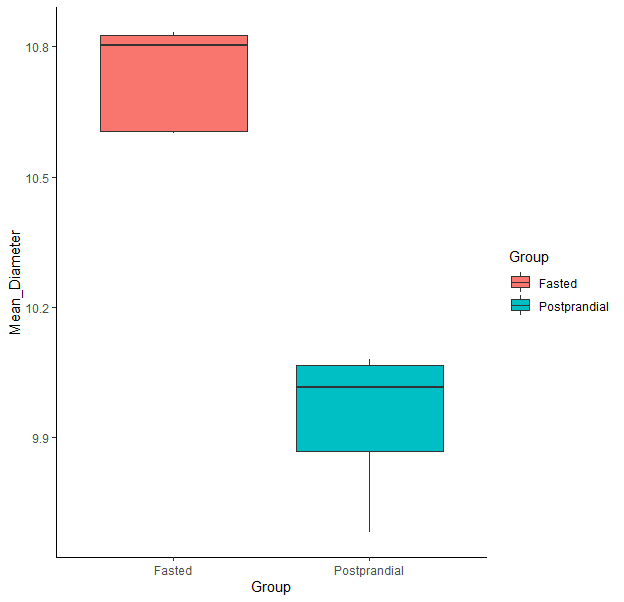


Supplementary Figure 1. The mean diameter of fasted HDL is larger than the mean diameter of postprandial HDL.


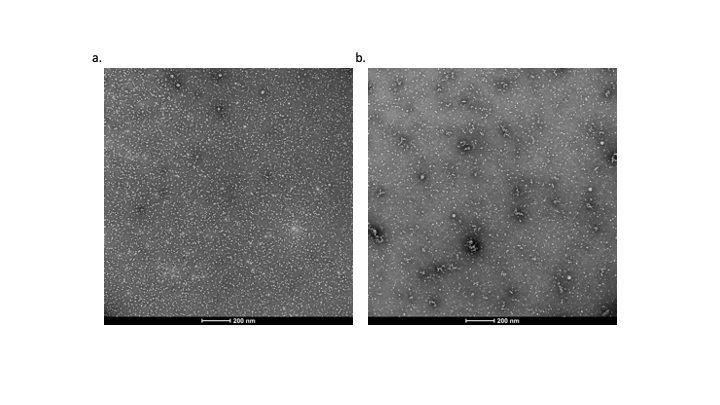


Supplementary Figure 2. Transmission Electron microscopy images of isolated HDL particles from 36-hour fasting study at a) at postprandial state (timepoint b), and b) at 36-hour fasted state (timepoint c).


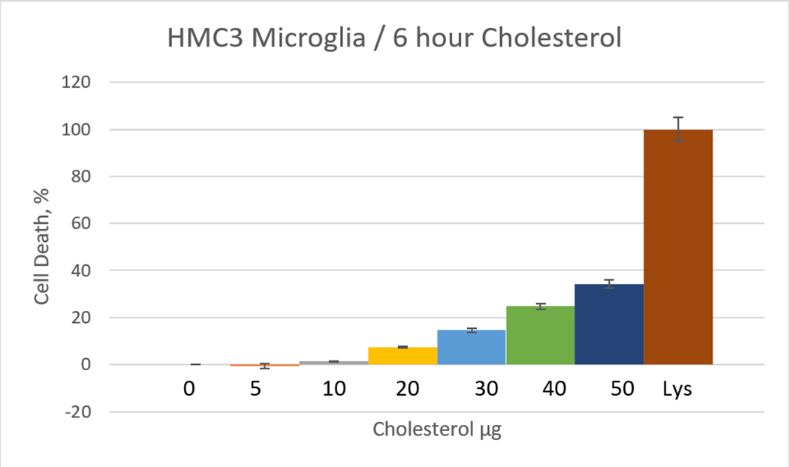


a


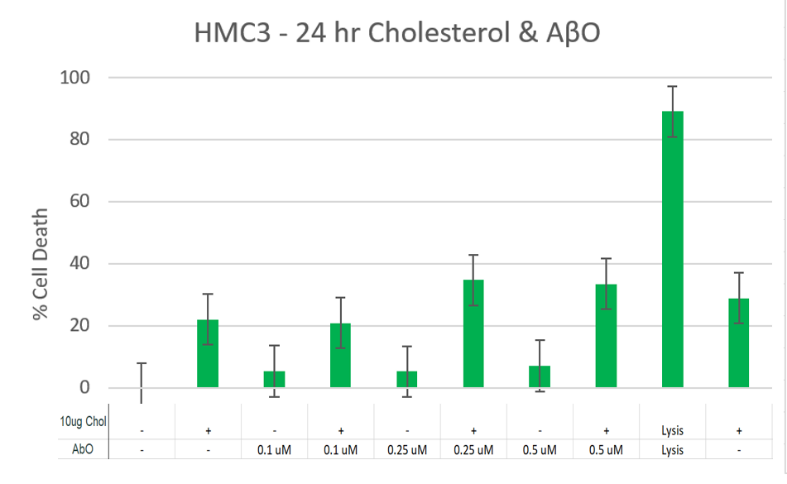


c

b

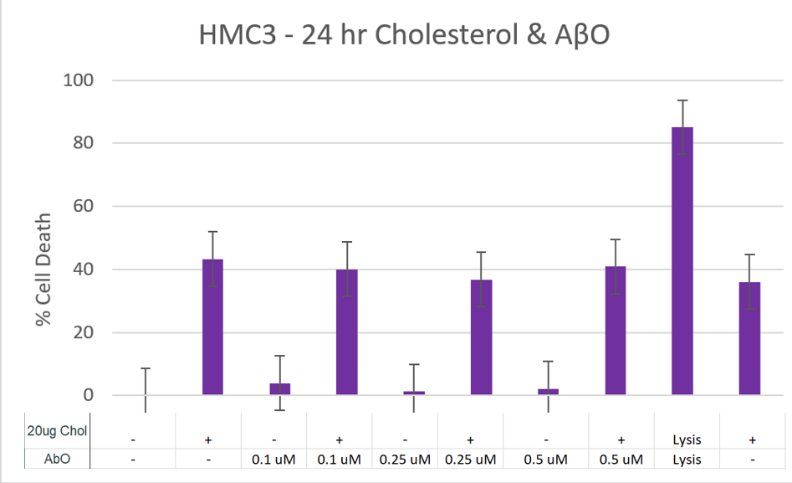


Supplementary Figure 3. HMC3 Cytotoxicity Assay. A) Cholesterol loaded HMC3 microglia (varying doses), for 6 hours. B) Cholesterol (10µg/mL) loaded and AβO treated HMC3 microglia, for 24 hours. C) Cholesterol (20µg/mL) loaded and AβO treated HMC3 microglia, for 24 hours.
